# Supplementary material for: PCP auto count: a novel Fiji/ImageJ plug-in for automated quantification of planar cell polarity and cell counting
Source: Front Cell Dev Biol. 2024 May 17;12:1394031. doi: 10.3389/fcell.2024.1394031 (PMC11140036; doi:10.3389/fcell.2024.1394031)
Supplement: Supplementary file 1 [file DataSheet1.pdf]

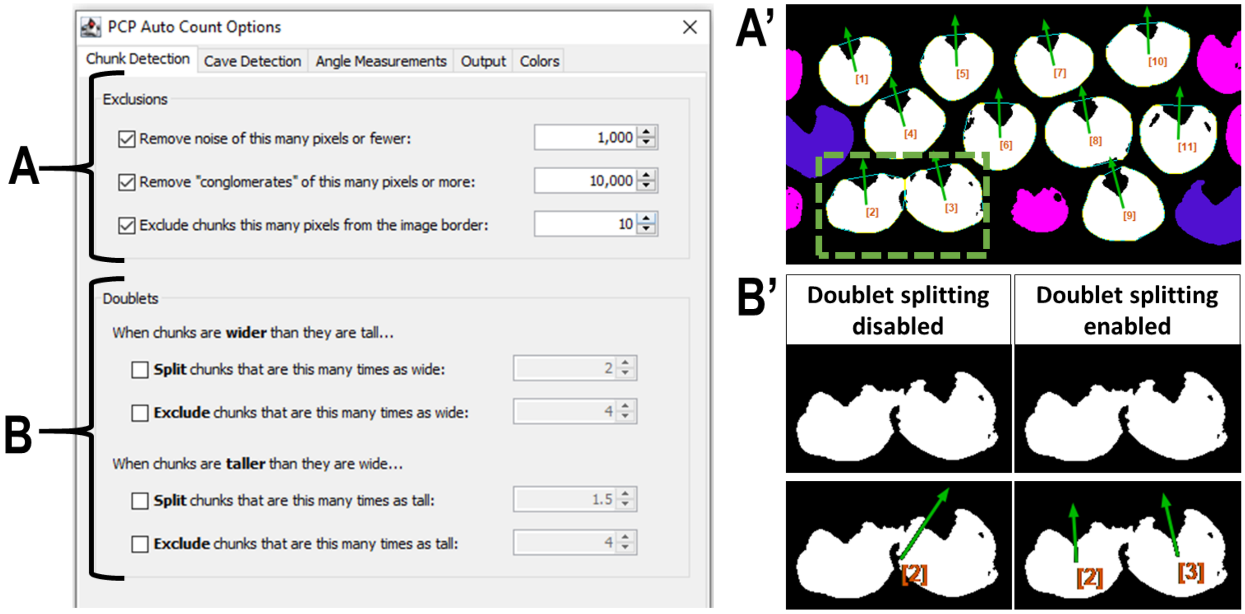

Supplementary Figure S1. User option menu for PCPA chunk detection. **(A)** The exclusion criteria box allows users to disregard chunks smaller and/or larger than a specified pixel size, and to exclude cells a set pixel distance from the image border. **(A')** Example output where size excluded chunks are shown as pink pseudo-coloring and border excluded chunks are shown as blue pseudo-coloring. **(B)** To address instances where two chunks touch after thresholding, users can set options to split chunks meeting a set ratio of wider-than-tall or taller-than-wide, or to exclude these chunks from further analyses. **(B')** Subset of A' (green box). If no doublet splitting options are applied, PCPA will treat this chunk as one mass and the angle measurements will incorrectly calculate based on the total chunk mass and dominant cave of the chunk. When doublet splitting options are applied, PCPA will split the doublet in half. The two resulting halves will be treated as independent chunks with chunk and cave centroid calculations being based on the mass contained within each respective box.

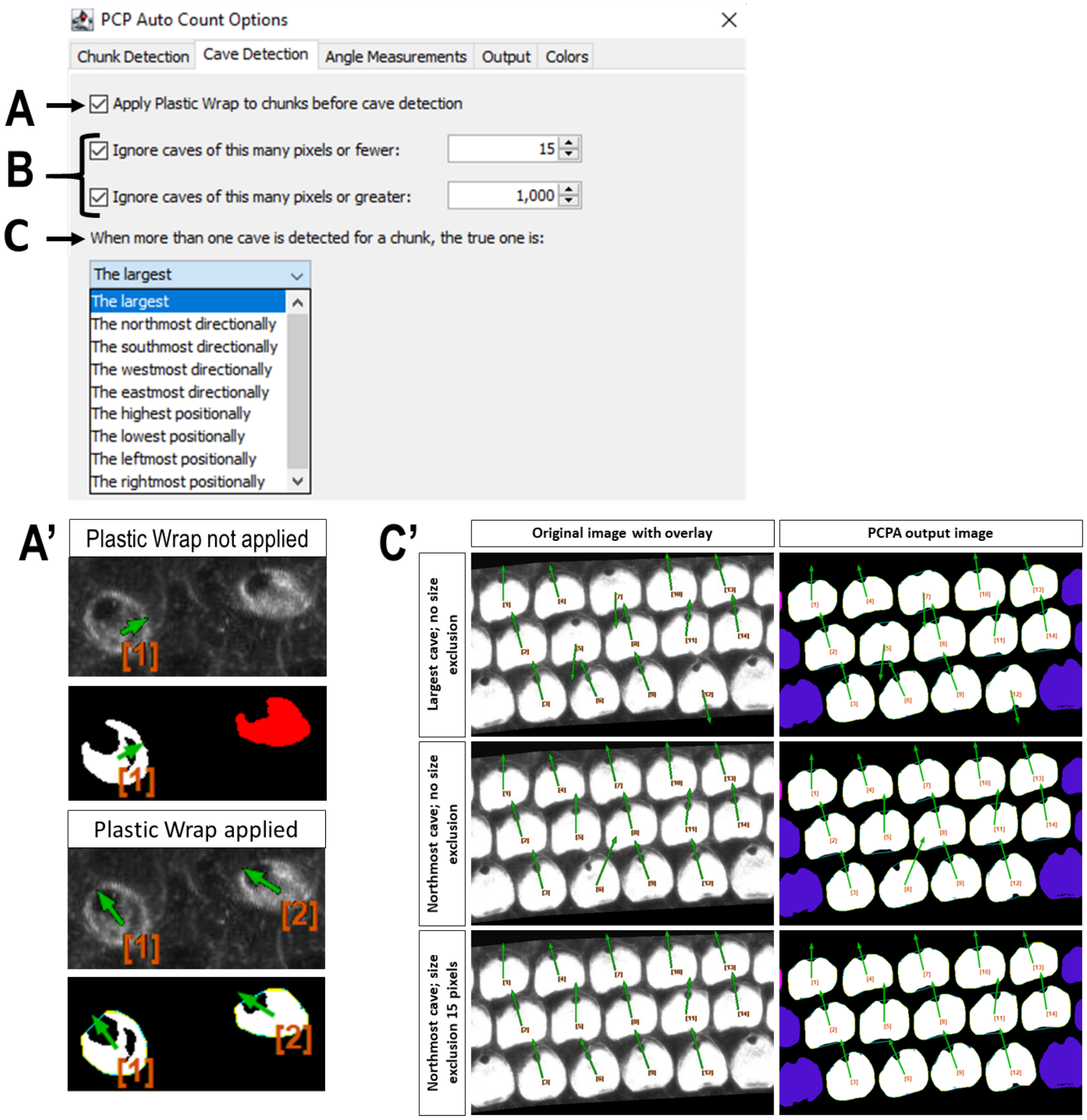

Supplementary Figure S2. User menu for PCPA cave detection. **(A)** Users are recommended to apply the Plastic Wrap function to their analyses. Plastic Wrap allows PCPA to group pixels that are proximal, but not abutting, into the same chunk. **(A')** Example images show that when plastic wrap is not applied, chunk 1 selects an incorrect cave, and chunk 2 is excluded from analysis for having no cave. When plastic wrap is applied, PCPA artificially closes off the open cave and selects the correct cave as the cave of interest. **(B)** Users can set minimum and maximum size requirements for cave of interest selection. **(C)** Users can set a directional requirement for cave of interest selection. Selecting a directional characteristic (e.g. northmost) will instruct PCPA to designate the cell's cave as the northmost inclusion whose size is between the upper and lower limits designated by the user. **(C')** Users can combine directional characteristics with upper or lower size limits for caves, in order to filter out incidental inclusions created by Plastic Wrap, pitting caused by the thresholding process, or large inclusions caused by irregular cell shapes.

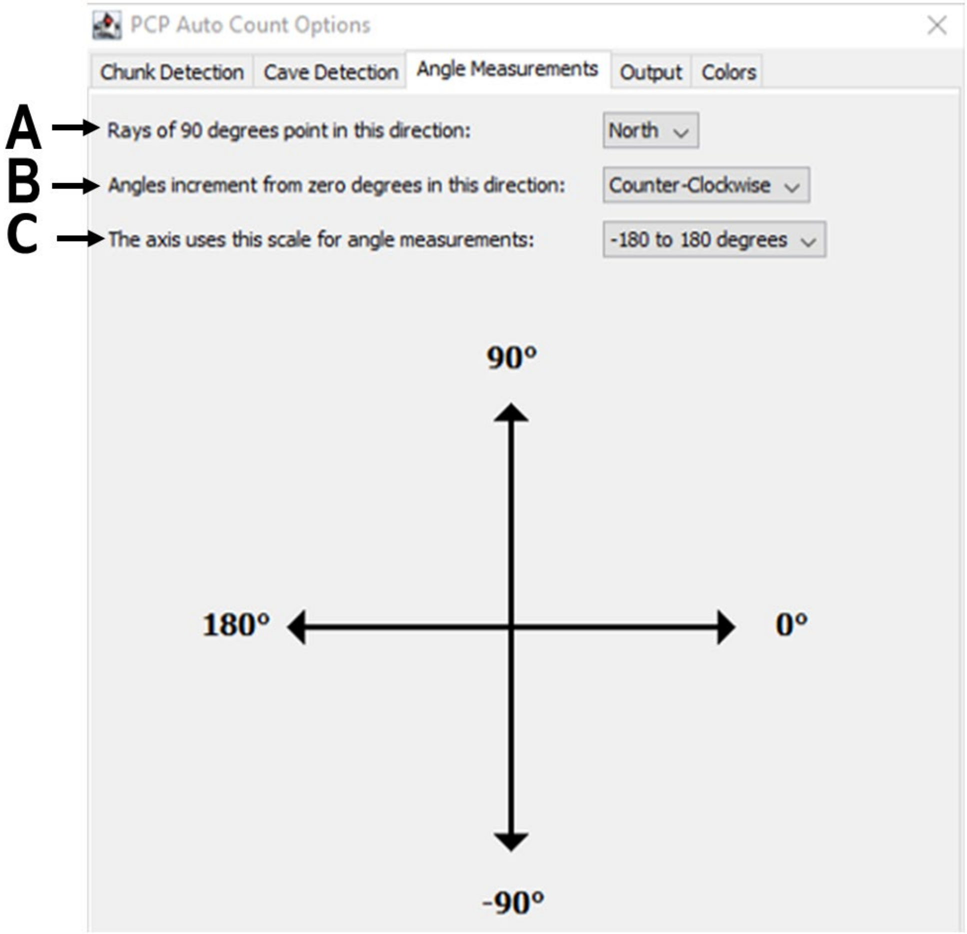

Supplementary Figure S3. User options for angle measurement calculations. **(A)** Option to set the direction of 90° ray. **(B)** Option to set direction of axis incrementation. **(C)** Option to set the scale of the (XY) axis to -180°/180° or 0°/360°

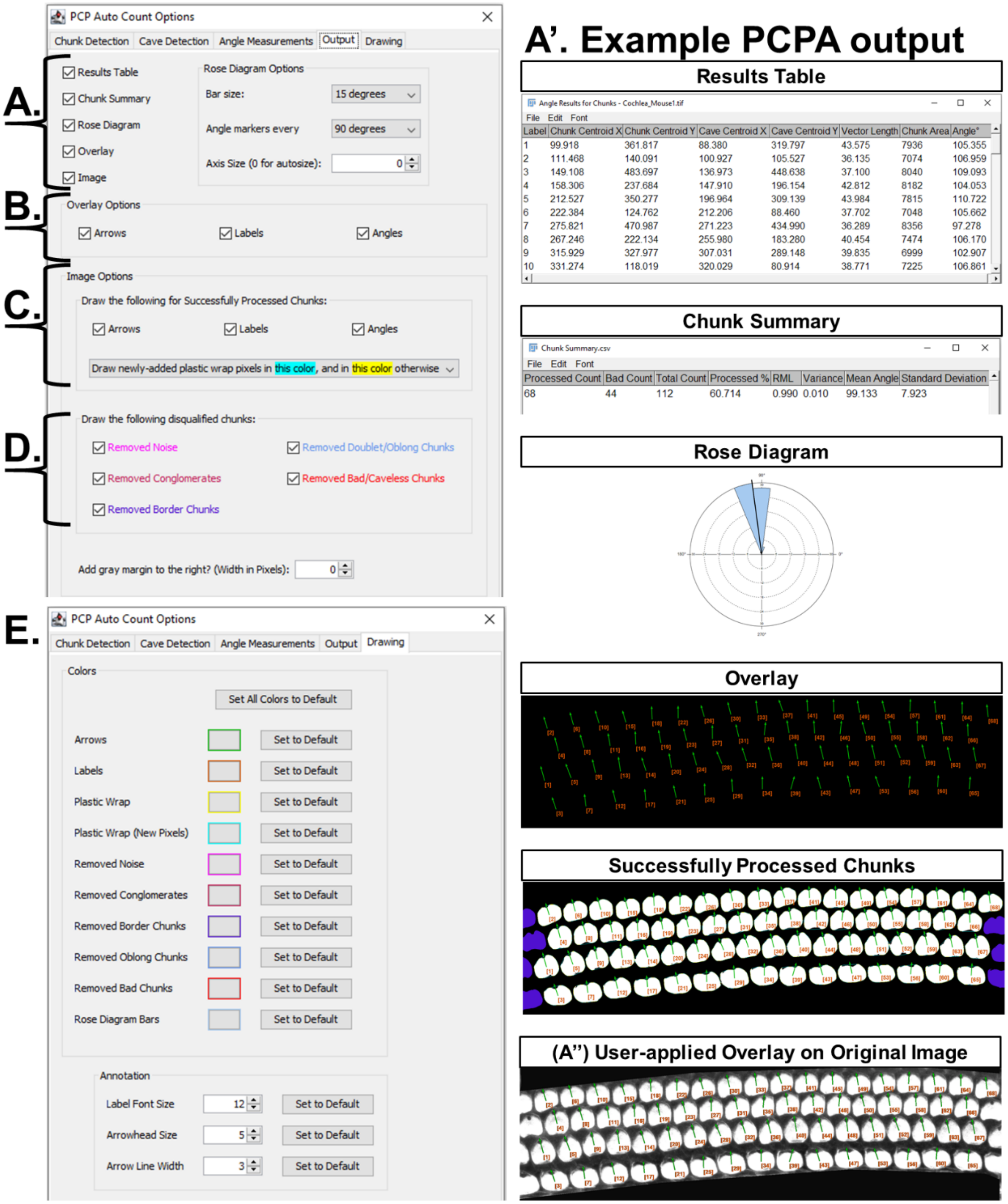

Supplementary Figure S4. PCPA data output tab. **(A)** Options for data metrics produced by PCPA. **(A')** Example of data metrics in A. **(A'')** Example of overlay superimposed on original preprocessed image. **(B)** The overlay (and selected overlay information) can be superimposed over the original image for data visualization. **(C)** Optional labels can be viewed on the post-PCPA image. **(D)** Color coding of the post-PCPA image can indicate to the investigator what chunks may have been excluded and why. **(E)** The Drawing tab allows users to customize color coding options from D as well as change font size and arrow thickness for output overlays.

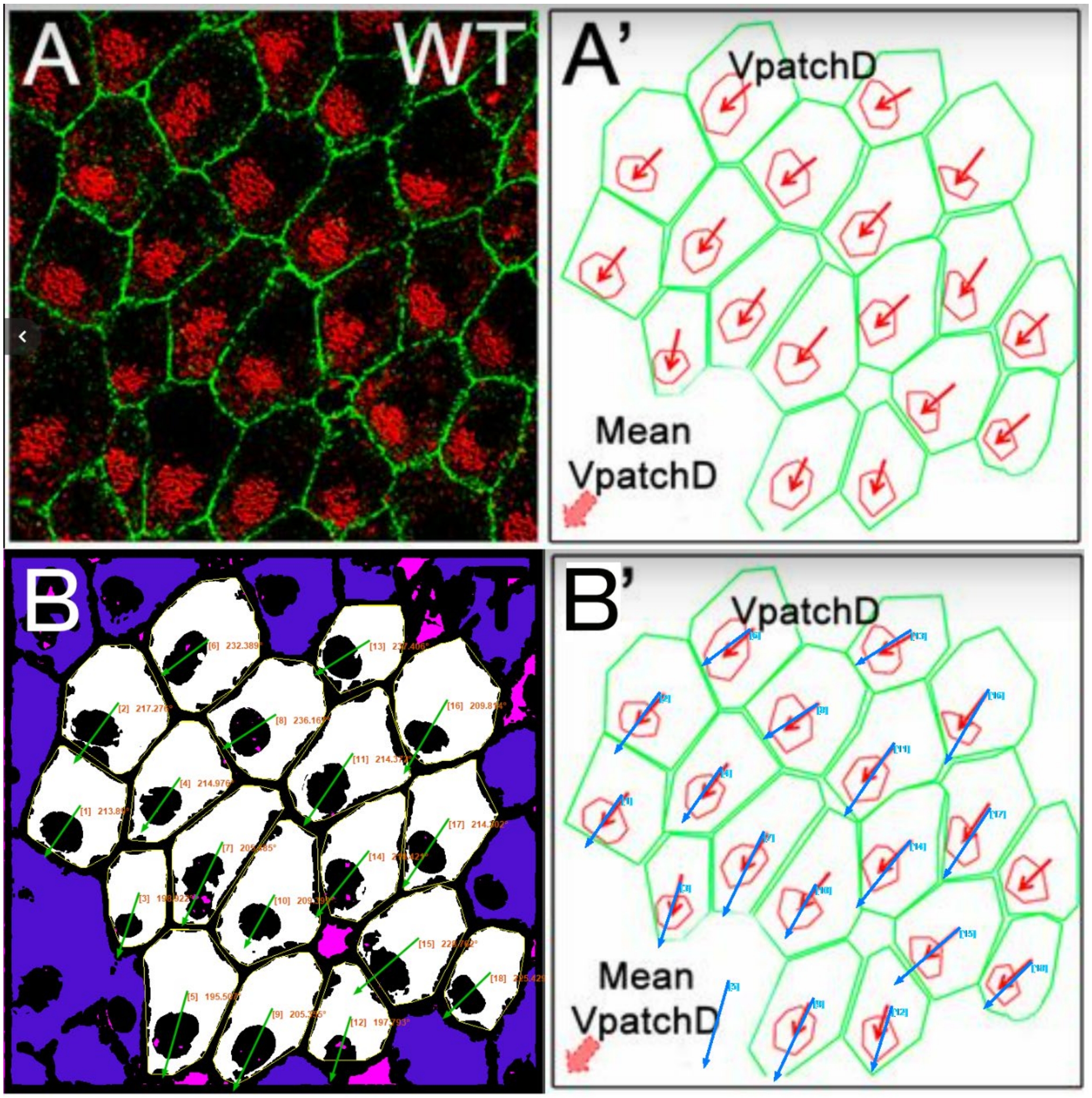

Supplementary Figure S5. PCPA recapitulates angle measurements taken from WT murine ependymal cells. Panels (A) and (A') are reprinted from *Proc. Natl. Acad. Sci.*, 111 , Boutin et al., A dual role for planar cell polarity genes in ciliated cells, Pages no. E3129-E3138, Copyright (2014), with permission from PNAS.. **(A)** A confocal image of WT mouse ependymal cells labeled with antibodies against ZO-1 (green) and γ-tubulin (red). **(A')** Manual tracings (green and red) of the cells in (A) with overlaid angle measurement arrows (red), as originally shown in Boutin et al. (Boutin et al., 2014). **(B)** Annotated output of image (A) after preprocessing and data collection with PCPA. The annotated output shows Plastic Wrap (yellow), edge excluded chunks (blue), size excluded chunks (pink), cell ID number with angle measurement (orange text), and vector arrows (green). **(B')** Arrows from the PCPA analysis (blue) are overlaid onto the image from (A') to show the high level of agreement between PCPA and the angle measurements reported by Boutin et al. (Boutin et al., 2014).

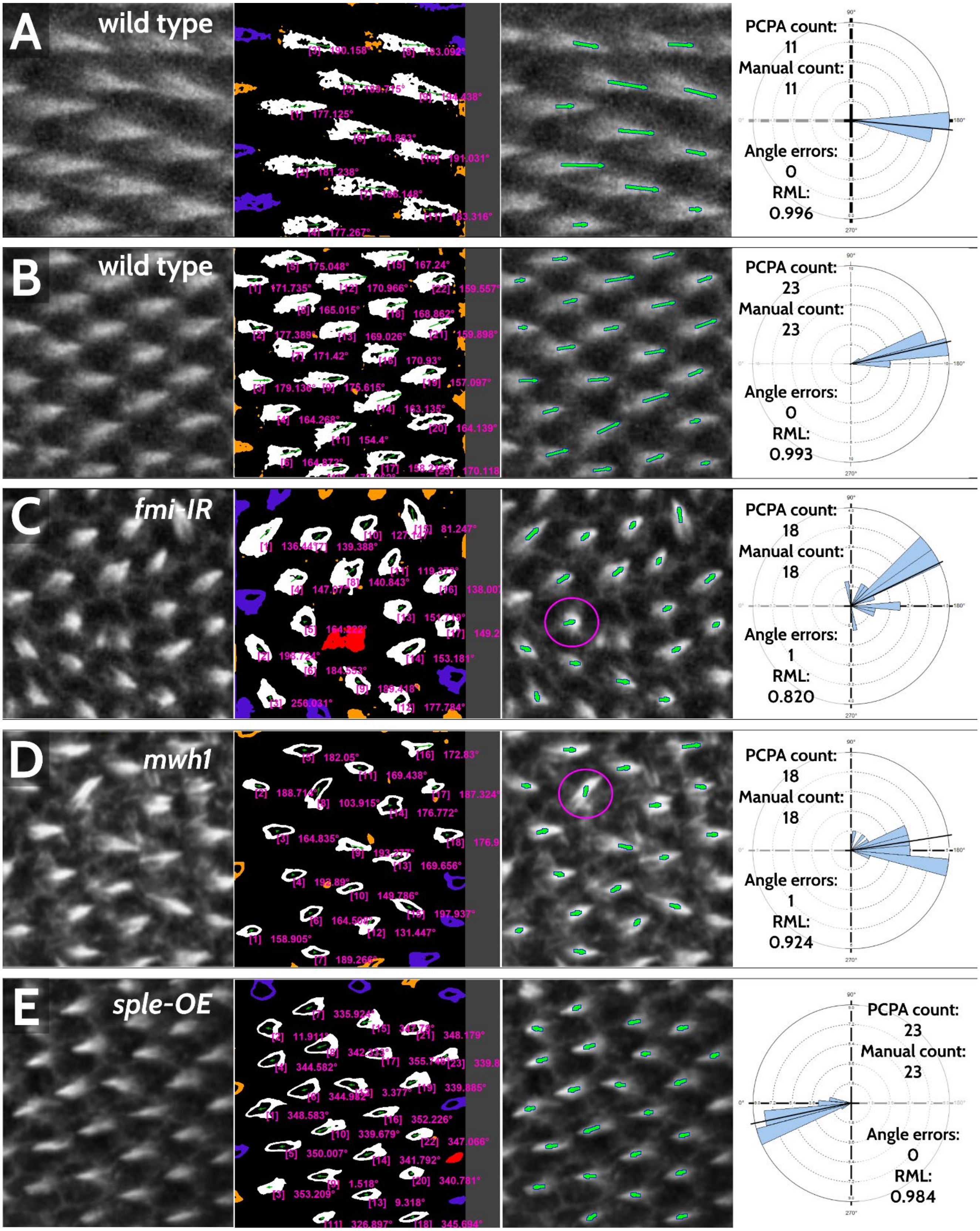
A
wild type
PCPA count: 11
Manual count: 11
Angle errors: 0
RML: 0.996
B
wild type
PCPA count: 23
Manual count: 23
Angle errors: 0
RML: 0.993
C
fmi-IR
PCPA count: 18
Manual count: 18
Angle errors: 1
RML: 0.820
D
mwh1
PCPA count: 18
Manual count: 18
Angle errors: 1
RML: 0.924
E
sple-OE
PCPA count: 23
Manual count: 23
Angle errors: 0
RML: 0.984

Supplementary Figure S6. PCPA accurately counts and measures Drosophila wing hairs (trichomes) labeled by phalloidin. Images reprinted from *Nat Commun*, 7(11135), Carvajal-Gonzalez, Roman, and Mlodzik, Positioning of centrioles is a conserved readout of Frizzled planar cell polarity signaling, Copyright (2016), with permission under CC-4.0. The leftmost column consists of original images from (Carvajal-Gonzalez et al., 2016). The next column shows PCPA angle measurements and output from thresholded trichomes. The third column shows original images overlaid with PCPA derived directional arrows. Images with overlaid arrows were analyzed, post-hoc, by a human experimenter to assess the accuracy of counts and angle measurements. The rightmost column shows rose diagrams generated for each image and lists the PCPA count, the human (manual) count, the number of PCPA angle measurements deemed incorrect by the human experimenter, and the resultant mean length (RML) of the measurements generated by PCPA for each image. For all images **(A-E)** the number of trichomes counted by PCPA and the human experimenter matched exactly. Of these 93 trichomes counted, PCPA determined angle measurements for 92 and the human experimenter rated 90 of these as accurate. **(A-B)** Wildtype *Drosophila* wing hairs measured by PCPA were rated as accurate by post-hoc human appraisal. **(C)** Fmi-IR (knockdown of the CELSR homolog Flamingo) Drosophila wing hairs measured by PCPA were rated by post-hoc observation as accurate, with the exception of one cell (indicated in magenta). However, due to the poorly defined morphology of the indicated cell, the human experimenter noted it would be difficult to manually measure this cell as well. **(D)** Multiple Wing Hairs 1 (mwh1) loss of function Drosophila wing hairs measured by PCPA were rated by post-hoc observation as accurate, with the exception of one cell (circled in magenta) that appears to be inaccurate by > 10 degrees. (E) Prickle/spiny-legs overexpressing (sple-OE) Drosophila wing hairs measured by PCPA were rated by post-hoc observation as accurate.

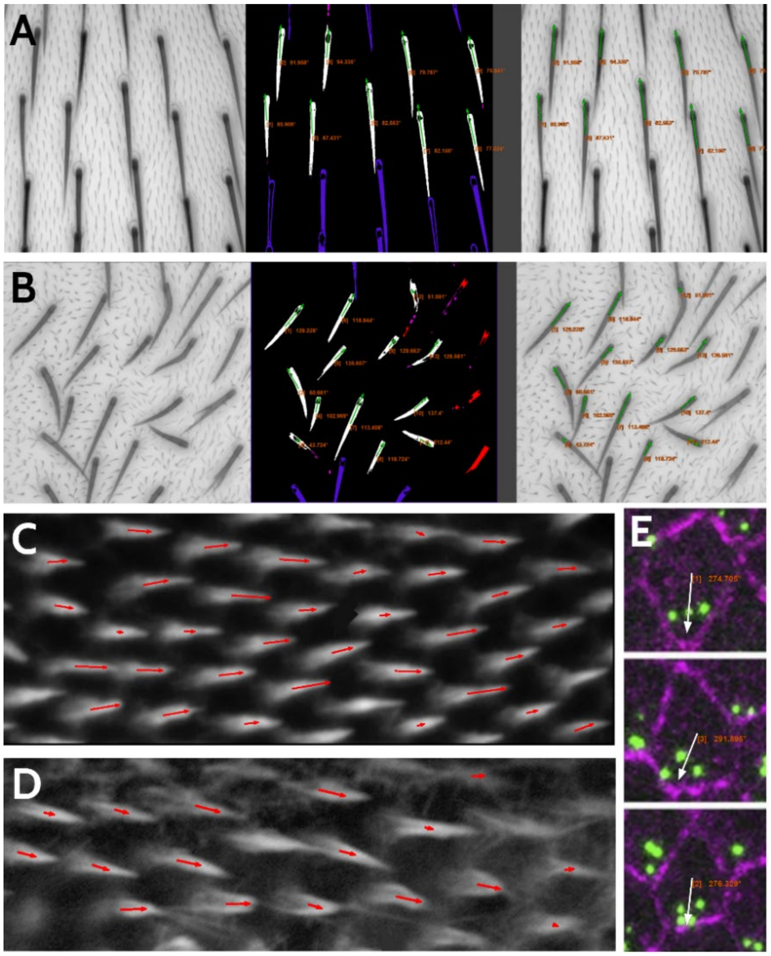

Supplementary Figure S7. PCPA can measure polarity via chunk and cave morphology across a variety of stained and unstained samples. Panels (A-D) are reprinted from *PLOS ONE*, 10(3), Lu and Adler, The diaphanous Gene of Drosophila Interacts Antagonistically with multiple wing hairs and Plays a Key Role in Wing Hair Morphogenesis, Copyright (2015), with permission under CC-4.0. **(A)** A brightfield image of trichomes on the thorax of Drosophila melanogaster (left) was thresholded and analyzed using PCPA (center). PCPA measurement arrows (green) overlaid onto the original image (right) were analyzed post-hoc by a human rater as accurate. (B) A brightfield image of thorax trichomes from a *Drosophila fuzzy* mutant (left) was thresholded and analyzed using PCPA (center). PCPA measurement arrows (green) overlaid onto the original image (right) were analyzed post-hoc by a human rater as accurate. **(C-D)** Micrographs of fluorescently conjugated phalloidin-labeled wing hairs (trichomes) from *Drosophila melanogaster* were measured using PCPA. Orientations identified by PCPA are shown as overlays of red arrows, and post-hoc analysis by a human rater found PCPA measurements to be accurate. Panels in (E) are reprinted from *Nat Commun*, 7(11135), Carvajal-Gonzalez, Roman, and Mlodzik, Positioning of centrioles is a conserved readout of Frizzled planar cell polarity signaling, Copyright (2016), with permission under CC-4.0. **(E)** Fluorescence-based micrographs of a *Drosophila* wing disk showing a GFP reporter for *Asl* (green) with immunolabeled flamingo (magenta). Overexpression of Asl led to many cells exhibiting multiple centrioles. These images could be thresholded such that the GFP of the multiple centrioles in each cell were merged to form a single cave and an inversion of the fmi fluorescence provided the boundaries of the chunks. Resultant PCPA measured angles are shown as overlays on the images and rated by a post-hoc human observer as accurate.

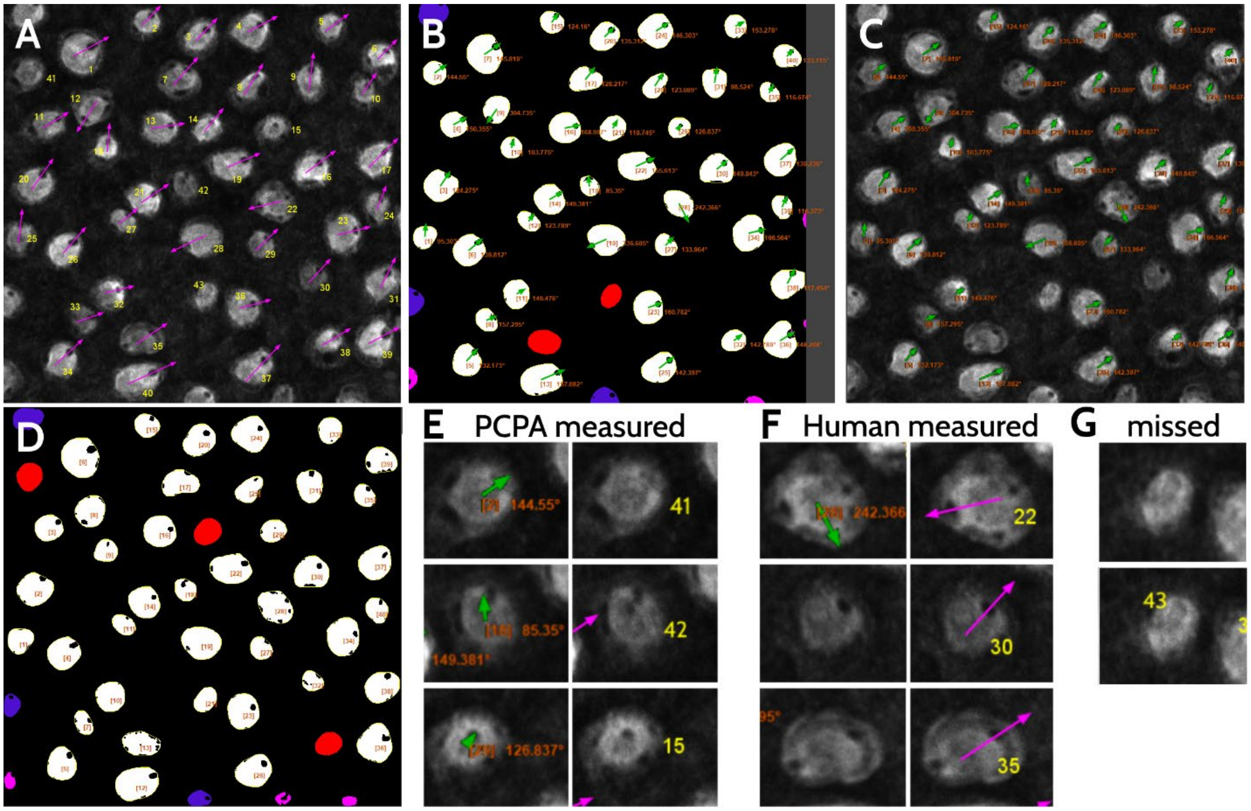

Supplementary Figure S8. Beta-spectrin labeling of hair cells in the adult mouse saccule presents challenges for both manual and automated PCP measurements. While most assessments of PCP phenotypes are conducted using tissues obtained during an animal's development, the adult mouse vestibular tissues, which have more variable quality of beta-spectrin labeling than tissue from younger animals, present an opportunity to challenge PCPA and compare its performance to human experimenters. **(A)** A representative image from a 60 day old C57Bl/6 mouse saccule immunostained with anti-βII-spectrin. A human experimenter with experience measuring vestibular PCP drew arrows (magenta) over all of the cells for which angles could be confidently measured, and numbered the cells (yellow) to obtain a cell count (n = 43). **(B)** PCPA output from the same original image as used for the analysis in (A). **(C)** PCPA overlay added to the non-thresholded image. **(D)** βII-spectrin labeling in adult saccules is of heterogeneous intensity; this required re-thresholding the image with slightly different parameters in order to obtain an accurate total cell count of n = 43. Thus, one of the challenges adult saccules present (and possibly other images with heterogeneous fluorescence) is that PCP angles and cell counts may not always be accomplished simultaneously with 100% accuracy and users may have to prioritize one or the other, or run two separate thresholded images through PCPA. To compare performance of the human experimenter versus PCPA, a third investigator examined the images and scored PCPA's performance for agreement on a 1-5 scale. While the majority of the angle measurements showed a high level of similarity between the human experimenter and PCPA, there were errors from both. **(E)** From this sample, PCPA was able to obtain angle measurements from 3 cells that the human experimenter did not feel confident to measure. The third investigator scored these PCPA measurements as 0, 1, and 0 (from top to bottom). **(F)** The human experimenter was able to measure PCP angles from 3 cells that PCPA either excluded or measured incorrectly (defined as a score of 2 or higher by the third investigator). Interestingly, the third investigator was not able to confidently score the human measured angle for the first cell (#22), but was confident the PCPA arrow was incorrect as all

the other cells in the image either point toward the upper right corner or the lower left corner, whereas the PCPA arrow points to the lower right corner. The third investigators gave scores of 0 and 0 for the two other cells in (F). These images highlight another challenge with βII-spectrin labeling in adult tissues: the presence of multiple β-spectrin-negative "caves" present on the apical surfaces of the cells. **(G)** Both the human experimenter and PCPA were able to count 43 cells in the image (excluding those touching the image border), with one cell in this sample excluded from angle analysis by both the human experimenter and by PCPA.

Supplementary Table S1

Materials and reagents used			
**Product**	**Company**	**Cat. number**	**RRID**
Anti-βII-spectrin	BD Biosciences	612562	AB_399853
Goat anti-Mouse IgG1 Cross-Adsorbed Secondary Antibody, Alexa Fluor™ 647	Thermo Fisher Scientific	A-21240	AB_2535809
Alexa Fluor™ 488 Phalloidin	Thermo Fisher Scientific	A12379	n/a
Hoechst 33342	Thermo Fisher Scientific	62249	AB_10626776
Image-iT™ FX Signal Enhancer	Thermo Fisher Scientific	I36933	n/a
M.O.M.® (Mouse on Mouse) Blocking Reagent	Vector Laboratories	MKB-2213-1	AB_2336587
16% Paraformaldehyde aqueous solution	Electron Microscopy Sciences	15710	n/a
Bovine serum albumin	Thermo Scientific Chemicals	J65097.22	n/a
Normal goat serum	Lampire Biological Laboratories	7322500	n/a
Triton X-100	MP Biomedicals 100 mL	ICN807423	n/a
Fluoro Gel with DABCO	Electron Microscopy Services	17985-02	n/a
R Project for Statistical Computing	R Core Team	n/a	SCR_001905
BioVoxxel Toolbox	BioVoxxel	n/a	RRID:SCR_015825
Fiji is just ImageJ	Fiji	n/a	RRID:SCR_002285

References

Boutin, C., Labedan, P., Dimidschstein, J., Richard, F., Cremer, H., André, P., et al. (2014). A dual role for planar cell polarity genes in ciliated cells. *Proc. Natl. Acad. Sci. U.S.A.* 111. doi: 10.1073/pnas.1404988111

Carvajal-Gonzalez, J. M., Roman, A.-C., and Mlodzik, M. (2016). Positioning of centrioles is a conserved readout of Frizzled planar cell polarity signalling. *Nat Commun* 7, 11135. doi: 10.1038/ncomms11135

Lu, Q., and Adler, P. N. (2015). The diaphanous Gene of Drosophila Interacts Antagonistically with multiple wing hairs and Plays a Key Role in Wing Hair Morphogenesis. *PLoS ONE* 10, e0115623. doi: 10.1371/journal.pone.0115623
